# Twenty-Year Benefit From Adjuvant Goserelin and Tamoxifen in Premenopausal Patients With Breast Cancer in a Controlled Randomized Clinical Trial

**DOI:** 10.1200/JCO.21.02844

**Published:** 2022-07-21

**Authors:** Annelie Johansson, Huma Dar, Laura J. van ’t Veer, Nicholas P. Tobin, Gizeh Perez-Tenorio, Anna Nordenskjöld, Ulla Johansson, Johan Hartman, Lambert Skoog, Christina Yau, Christopher C. Benz, Laura J. Esserman, Olle Stål, Bo Nordenskjöld, Tommy Fornander, Linda S. Lindström

**Affiliations:** ^1^ Department of Oncology and Pathology, Karolinska Institutet, Stockholm, Sweden; ^2^ Department of Laboratory Medicine, University of California San Francisco, San Francisco, CA; ^3^ Department of Biomedical and Clinical Sciences and Department of Oncology, Linköping University, Linköping, Sweden; ^4^ Institution of Clinical Sciences, Department of Oncology, Sahlgrenska Academy at Gothenburg University, Gothenburg, Sweden; ^5^ Oncological Centre, Karolinska University Hospital, Stockholm, Sweden; ^6^ Buck Institute for Research on Aging, Novato, CA; ^7^ Department of Surgery, University of California San Francisco, San Francisco, CA; ^8^ Department of Medicine, University of California San Francisco, San Francisco, CA

## Abstract

To assess the long-term (20-year) endocrine therapy benefit in premenopausal patients with breast cancer.

**METHODS:**

Secondary analysis of the Stockholm trial (STO-5, 1990-1997) randomly assigning 924 premenopausal patients to 2 years of goserelin (3.6 mg subcutaneously once every 28 days), tamoxifen (40 mg orally once daily), combined goserelin and tamoxifen, or no adjuvant endocrine therapy (control) is performed. Random assignment was stratified by lymph node status; lymph node–positive patients (n = 459) were allocated to standard chemotherapy (cyclophosphamide, methotrexate, and fluorouracil). Primary tumor immunohistochemistry (n = 731) and gene expression profiling (n = 586) were conducted in 2020. The 70-gene signature identified genomic low-risk and high-risk patients. Kaplan-Meier analysis, multivariable Cox proportional hazard regression, and multivariable time-varying flexible parametric modeling assessed the long-term distant recurrence-free interval (DRFI). Swedish high-quality registries allowed a complete follow-up of 20 years.

**RESULTS:**

In estrogen receptor–positive patients (n = 584, median age 47 years), goserelin, tamoxifen, and the combination significantly improved long-term distant recurrence-free interval compared with control (multivariable hazard ratio [HR], 0.49; 95% CI, 0.32 to 0.75, HR, 0.57; 95% CI, 0.38 to 0.87, and HR, 0.63; 95% CI, 0.42 to 0.94, respectively). Significant goserelin-tamoxifen interaction was observed (*P* = .016). Genomic low-risk patients (n = 305) significantly benefitted from tamoxifen (HR, 0.24; 95% CI, 0.10 to 0.60), and genomic high-risk patients (n = 158) from goserelin (HR, 0.24; 95% CI, 0.10 to 0.54). Increased risk from the addition of tamoxifen to goserelin was seen in genomic high-risk patients (HR, 3.36; 95% CI, 1.39 to 8.07). Moreover, long-lasting 20-year tamoxifen benefit was seen in genomic low-risk patients, whereas genomic high-risk patients had early goserelin benefit.

**CONCLUSION:**

This study shows 20-year benefit from 2 years of adjuvant endocrine therapy in estrogen receptor–positive premenopausal patients and suggests differential treatment benefit on the basis of tumor genomic characteristics. Combined goserelin and tamoxifen therapy showed no benefit over single treatment. Long-term follow-up to assess treatment benefit is critical.

## INTRODUCTION

Premenopausal patients with breast cancer have an increased risk of fatal disease.^1-4^ Since these patients are diagnosed early in life, the long-term risk and treatment benefit are of particular interest in this patient group. Standard adjuvant treatment of premenopausal estrogen receptor (ER)–positive breast cancer is tamoxifen for 5 years or more.^5-7^ Additional ovarian function suppression (OFS), such as the luteinizing hormone-releasing hormone-agonist goserelin, and/or chemotherapy is recommended in high-risk disease, often defined by standard clinical markers including lymph node involvement, high tumor grade, high proliferation, high genomic risk signature scores, and an age below 40 years.^5-7^ Therefore, although clinicians are becoming more inclined to recommend OFS, it is not clear which patients who should be offered the treatment.^8,9^

**CONTEXT**

**Key Objective**
The long-term endocrine therapy benefit in premenopausal patients with breast cancer, diagnosed early in life, is important to understand, given the long-term risk of distant recurrence in estrogen receptor (ER)–positive disease. The unique STO-5 trial with a complete follow-up of 20 years randomly assigned premenopausal patients to adjuvant goserelin, tamoxifen, combined goserelin-tamoxifen therapy, or no endocrine therapy (control). To our knowledge, for the first time the long-term benefit of goserelin and tamoxifen in premenopausal patients is assessed.
**Knowledge Generated**
This study demonstrates long-term benefit from 2 years of adjuvant endocrine therapy in ER-positive premenopausal patients. Furthermore, it suggests long-lasting benefit from tamoxifen in genomic low-risk patients with long-term risk of distant recurrence, whereas genomic high-risk patients have early risk and benefit from goserelin.
**Relevance**
Premenopausal patients with ER-positive breast cancer have long-term benefit of endocrine therapy; however, the heterogenous metastatic potential gives rise to differential treatment benefit and a need for personalized endocrine therapy.

Results from the Suppression of Ovarian Function Trial (SOFT) suggested that addition of OFS to tamoxifen significantly improves survival in premenopausal patients, with a relatively short median follow-up of 8 years.^10,11^ However, random assignment to OFS only was not included in SOFT, and since patient enrollment was in the early 2000s, random assignment to an untreated group was not possible. Moreover, subanalysis concluded that the main effect from combined tamoxifen and OFS was seen in a group of slightly younger (median age 40 years) clinically high-risk patients who had regained their premenopausal status after prior chemotherapy.^10,11^ Contrary to SOFT, the Zoladex in Premenopausal Patients (ZIPP) trial suggested that combined goserelin and tamoxifen therapy is not superior to either modality alone.^12^ It might seem controversial that a combined treatment is not better than single treatment, but this was also seen in the Arimidex, Tamoxifen Alone or in Combination (ATAC) study in postmenopausal patients, where the combination of aromatase inhibitor and tamoxifen did not improve survival compared with aromatase inhibitor alone.^13^ A strength of the Stockholm part of the ZIPP trial (STO-5) is that patients were strictly randomly assigned to the four trial arms, and therefore, the STO-5 trial has also been analyzed separately.^14-19^ Furthermore, since the premenopausal endocrine therapy benefit was not clearly established at trial start, random assignment to no adjuvant endocrine therapy was included in the STO-5 trial. Thus, this four-arm trial now enables unique assessment of endocrine therapy versus control, in combination with unique long-term follow-up from high-quality Swedish registries.

Ample evidence shows that patients with ER-positive breast cancer have a steady long-term risk of developing distant metastatic recurrences, with a large proportion of these events occurring beyond 10 years after primary diagnosis.^20-26^ Given this, longer follow-up is needed to understand the true endocrine treatment benefit in ER-positive breast cancer. In addition, the long-term benefit of adjuvant OFS therapy remains unknown, as there is a general lack of clinical trials with 10 or more years outcome data on this. The STO-5 trial has now reached a complete follow-up of 20 years; here, we present the long-term endocrine therapy benefit in premenopausal patients with breast cancer randomly assigned to two years of goserelin, tamoxifen, the combination of the two, or no adjuvant endocrine therapy. Moreover, we assess treatment arm–specific endocrine therapy benefit according to genomic risk stratification, using the molecular 70-gene signature risk prediction tool.^27^ This signature has known prognostic utility among young patients with breast cancer,^28,29^ but whether it has endocrine therapy predictive value remains unexplored.

## METHODS

### Patients

**FIG 1.**
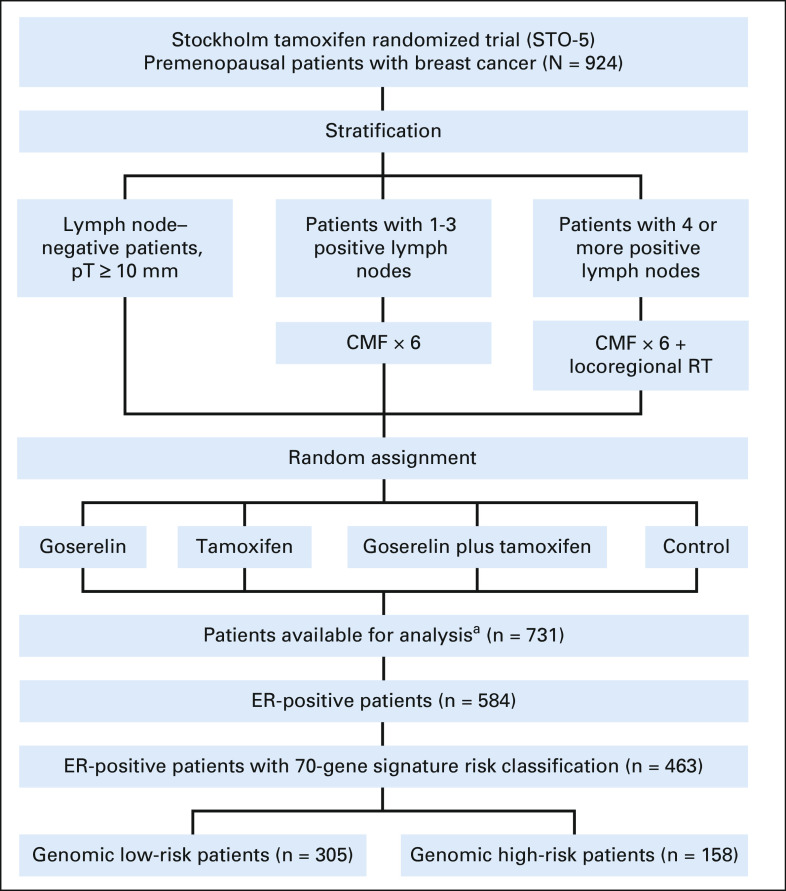
CONSORT diagram of the STO-5 trial. ^a^Seven hundred thirty-one patients with immunohistochemistry assessment. CMF, cyclophosphamide, methotrexate, and fluorouracil; ER, estrogen receptor; RT, radiotherapy.

From May 1990 to January 1997, the Stockholm trial (STO-5) enrolled 924 premenopausal patients diagnosed with invasive operable breast cancer.^14-19^ Random assignment included four trial arms: 2 years of goserelin (3.6 mg subcutaneously once every 28 days), tamoxifen (40 mg orally once daily), combination of goserelin and tamoxifen, or no adjuvant endocrine therapy (control). Random assignment was stratified in three groups on the basis of patients' lymph node status; 0, 1-3, and 4 or more positive lymph nodes (Fig 1). Concurrent with endocrine therapy, lymph node–positive patients received standard adjuvant chemotherapy (cyclophosphamide, methotrexate, and fluorouracil). Patients with four or more positive lymph nodes received locoregional radiotherapy; details are given in the Data Supplement, online only.

Informed consent was obtained before random assignment, and the trial was approved by the Karolinska Institutet Regional Ethics Committee. The trial was approved and initiated before the practice of trial registration in Sweden. The trial center was the Stockholm Regional Cancer Center.

Follow-up until December 31, 2016, was from Swedish high-quality national and regional registries of high validity and essentially complete coverage.^30-32^

### Immunohistochemistry

Immunohistochemical (IHC) analysis of ER, progesterone receptor (PR), human epidermal growth factor receptor 2 (HER2), and proliferation marker Ki-67 was performed in 2020 in collaboration with the Karolinska University Hospital Pathology Department following standardized clinical protocols; details are given in the Data Supplement. Analysis was conducted for 731 patients with available primary tumor formalin-fixed paraffin-embedded blocks with sufficient invasive tumor cells for analysis. The 731 patient subset had similar distribution of patient and tumor characteristics as the 924 originally enrolled patients (Data Supplement). The percentage of cancer cells positive for each marker was scored by experienced breast cancer pathologists. According to Swedish National Guidelines,^33^ ER-positive and PR-positive status was defined by a threshold of 10% or greater and Ki-67 was categorized as low (< 15%) and intermediate/high (≥ 15%). HER2 positivity was defined as intensity 3+ by IHC. ER status and PR status for patients with missing IHC data but with available immunoassay measurements^34,35^ (n = 4) were determined from this approach (Data Supplement). A total of 584 patients had ER-positive tumors (Fig 1).

### Tumor Size and Tumor Grade

Tumor size (T) was categorized into three groups according to clinical guidelines: T1a/b (≤ 10 mm), T1c (11-20 mm), and T2-T3 (> 20 mm). Tumor grading was performed according to the Nottingham Histologic Score system (Elston grade).^36^

### 70-Gene Signature Risk Classification

Agilent microarray gene expression profiling was performed in 2019-2021 and classified primary tumors into low or high genomic risk using the 70-gene signature; details are given in the Data Supplement.^27,37,38^ In total, 586 tumors passed the 70-gene signature quality check, whereof 463 were ER-positive (Fig 1).

### Statistical Analyses

The long-term endocrine therapy benefit was assessed by univariate Kaplan-Meier analysis and multivariable Cox proportional hazard regression. To assess how risk and treatment effect varied over the 20-year follow-up, multivariable time-varying analysis was conducted by flexible parametric survival modeling;^39^ details are given in the Data Supplement. End point was the distant recurrence-free interval (DRFI),^40,41^ including distant metastasis or fatal breast cancer (in patients with missing date of distant metastasis, n = 3) as the event. Adjustments included standard clinical patient and tumor characteristics (age, random assignment year, lymph node status, tumor size, tumor grade, PR status, HER2 status, Ki-67 status, and type of surgery [mastectomy or breast-conserving surgery]). Given trial stratification by lymph node status, this simultaneously adjusts for chemotherapy and locoregional radiotherapy. Interaction between goserelin and tamoxifen was tested by including a product term in the Cox proportional hazard regression model. All analyses were based on intention-to-treat. Analyses were performed in R version 3.5.2 and SAS version 9.4. All statistical tests were two-sided, and *P* values < .05 were considered statistically significant.

## RESULTS

Patient and tumor characteristics of the 584 ER-positive patients in the STO-5 trial were well-balanced between the four trial arms (Table 1). The median age was 47 (range, 26-55) years, and the majority of patients had grade 2 (63%), PR-positive (91%), HER2-negative (88%), and Ki-67–low (70%) tumors. According to the trial protocol, lymph node–positive patients (n = 301, 51%) received chemotherapy.

**TABLE 1.**
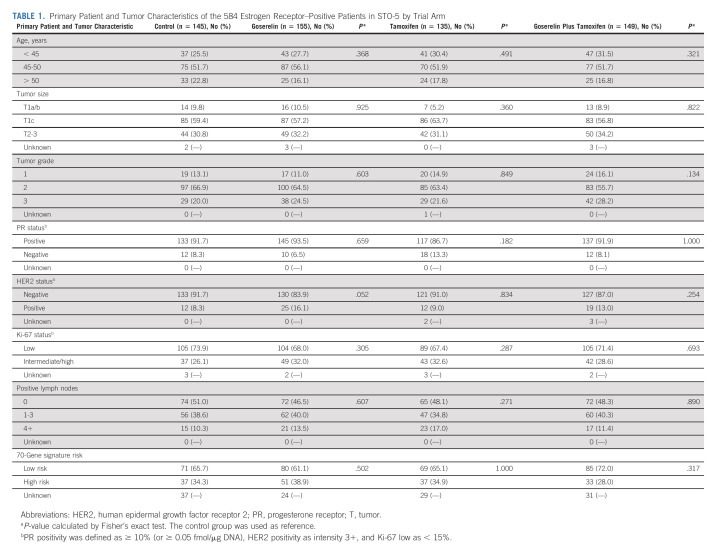
Primary Patient and Tumor Characteristics of the 584 Estrogen Receptor–Positive Patients in STO-5 by Trial Arm

### The Long-Term Endocrine Therapy Benefit by Trial Arm

**FIG 2.**
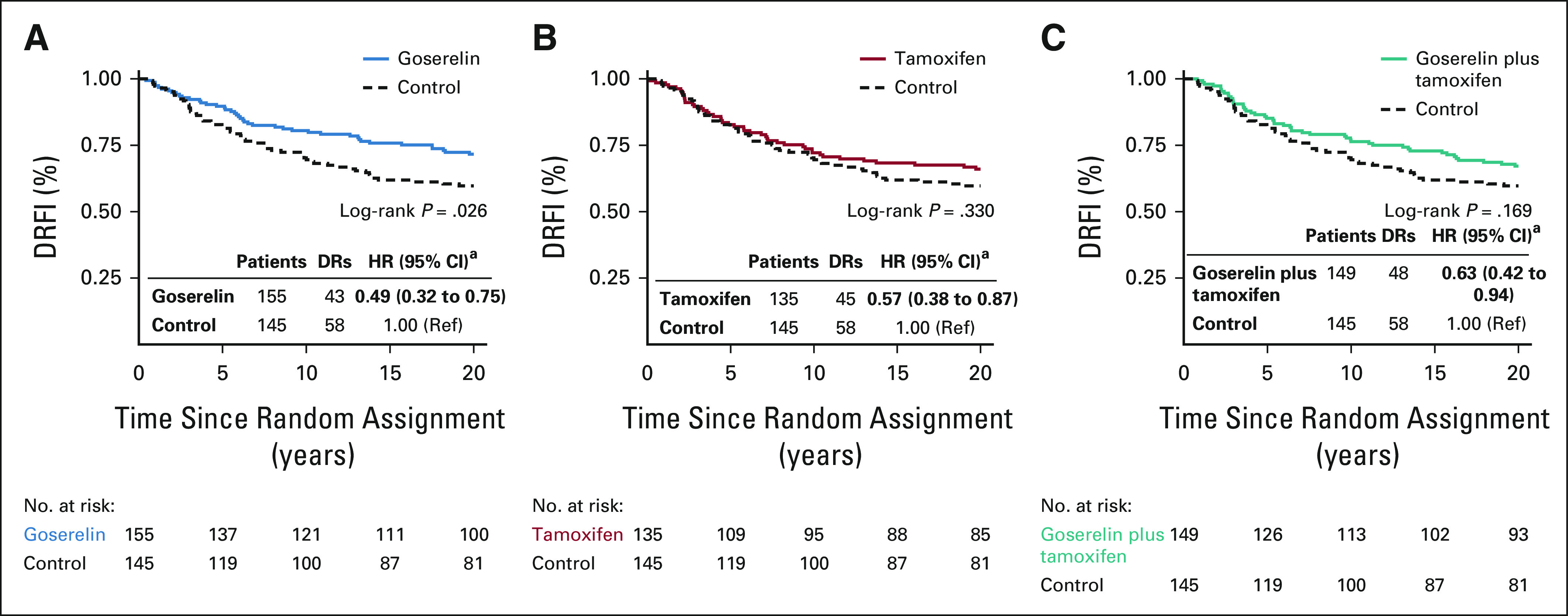
Kaplan-Meier and multivariable Cox proportional hazard regression analyses of DRFI in patients randomly assigned to 2 years of (A) goserelin, (B) tamoxifen, and (C) the combination of goserelin and tamoxifen, compared with patients randomly assigned to no adjuvant endocrine therapy (control). ^a^Multivariable analysis adjusted for age, random assignment year, lymph node status, tumor size, tumor grade, PR status, HER2 status, Ki-67 status, and type of surgery. DRFI, distant recurrence-free interval; DRs, distant recurrences; HER2, human epidermal growth factor receptor 2; HR, hazard ratio; PR, progesterone receptor; ref, reference.

Survival proportions at 20 years by DRFI were 71.6%, 66.0%, 67.1%, and 59.7% for patients randomly assigned to goserelin, tamoxifen, the combination, or control, respectively. Univariate Kaplan-Meier analysis showed significantly improved long-term DRFI from goserelin (log-rank *P* = .026, Fig 2A), compared with control. No significant difference in long-term DRFI was seen in univariate analysis from tamoxifen or the combination (Figs 2B and 2C). Multivariable Cox proportional hazard regression analysis showed significantly improved long-term DRFI from goserelin, tamoxifen, and the combination (hazard ratio [HR], 0.49; 95% CI, 0.32 to 0.75, HR, 0.57; 95% CI, 0.38 to 0.87, and HR, 0.63; 95% CI, 0.42 to 0.94, respectively), compared with control (Fig 2). Crude analysis adjusting for age, random assignment year, and lymph node status yielded similar estimates (Data Supplement).

Multivariable analyses also assessed the effect from goserelin in patients treated with or without tamoxifen and vice versa. No significant long-term benefit from the combination of goserelin and tamoxifen was seen, compared with tamoxifen only or goserelin only (Table 2). Furthermore, a significant interaction between goserelin and tamoxifen was observed (*P* = .016).

**TABLE 2.**
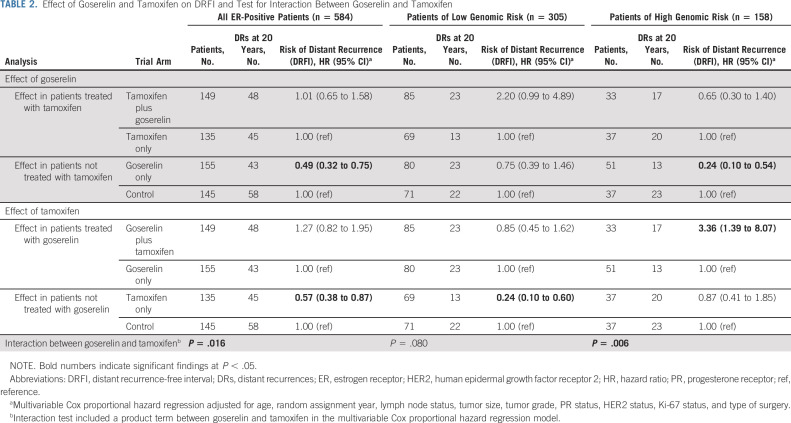
Effect of Goserelin and Tamoxifen on DRFI and Test for Interaction Between Goserelin and Tamoxifen

### The Long-Term Endocrine Therapy Benefit by Genomic Risk Stratification

**FIG 3.**
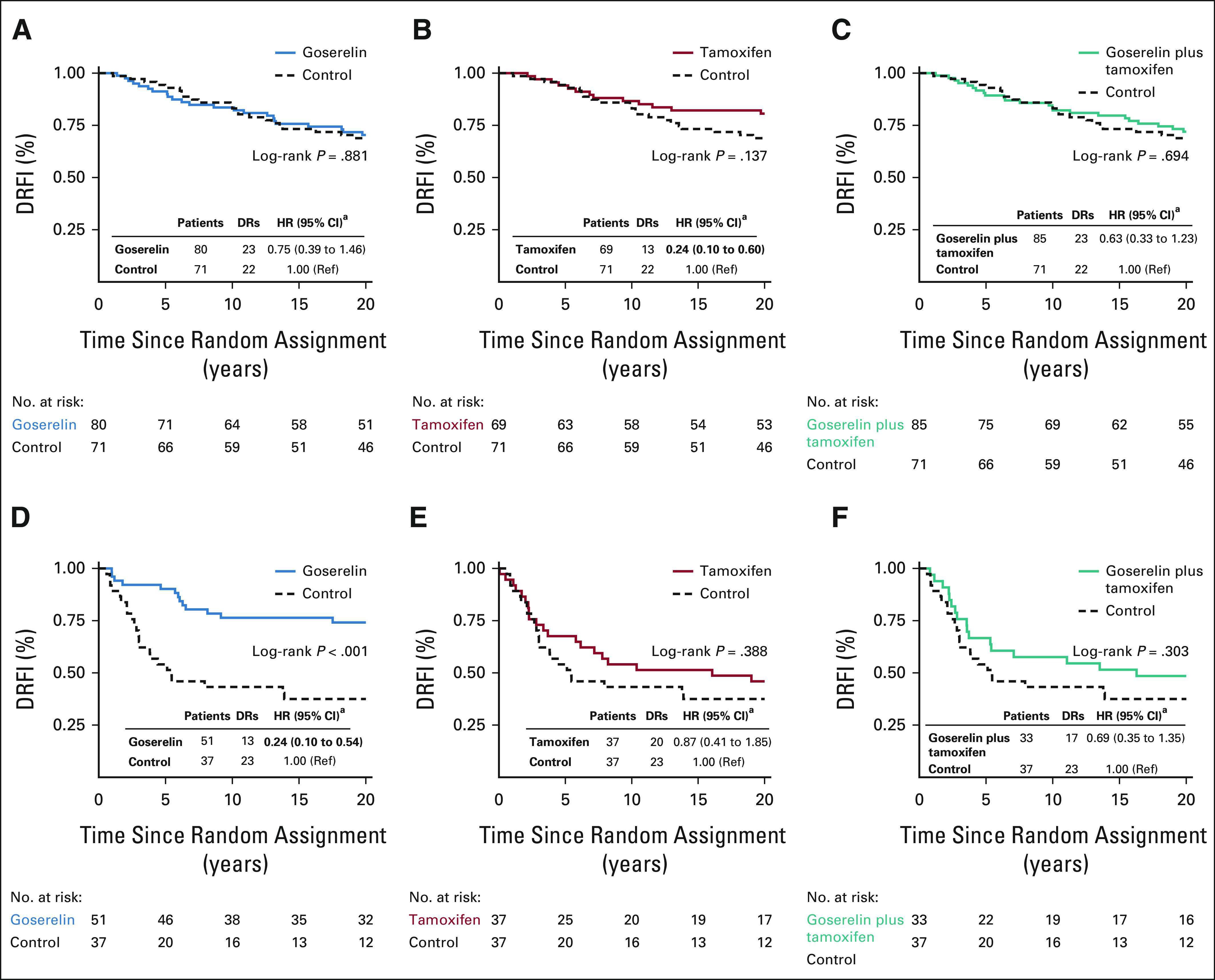
Kaplan-Meier and multivariable Cox proportional hazard regression analyses of DRFI in patients randomly assigned to 2 years of goserelin, tamoxifen, and the combination of goserelin and tamoxifen, compared with patients randomly assigned to no adjuvant endocrine therapy (control), stratified by genomic risk. (A-C) Patients of low genomic risk and (D-F) patients of high genomic risk. ^a^Multivariable analysis adjusted for age, random assignment year, lymph node status, tumor size, tumor grade, PR status, HER2 status, Ki-67 status, and type of surgery. DRFI, distant recurrence-free interval; DRs, distant recurrences; HER2, human epidermal growth factor receptor 2; HR, hazard ratio; PR, progesterone receptor; ref, reference.

The long-term endocrine therapy benefit was further assessed in the 463 ER-positive patient tumor samples with available 70-gene signature risk classification (Fig 3). The low number of patients with tumors classified as ultralow risk (n = 51) allowed no meaningful analyses. Genomic high-risk patients (n = 158) were more likely to be of younger age and lymph node–positive and have tumors of larger size, higher grade, PR-negative status, HER2-positive status, and higher Ki-67 levels (Data Supplement). Nevertheless, 60% and 47% of genomic high-risk and low-risk patients were lymph node–positive, respectively, and 37% of genomic high-risk patients were Ki-67–low.

Stratified univariate Kaplan-Meier analysis (Fig 3) showed no significantly improved long-term DRFI in genomic low-risk patients from tamoxifen (log-rank *P* = .137, Fig 3B), but significantly improved long-term DRFI was seen in genomic high-risk patients from goserelin (log-rank *P* < .001, Fig 3D), compared with control. Notably, in the Kaplan-Meier graphs, differences in DRFI from goserelin in genomic high-risk patients were observed early, whereas differences from tamoxifen in genomic low-risk patients were observed first after approximately 10 years (Figs 3B and 3D). Furthermore, stratified multivariable analyses showed significantly improved long-term DRFI from tamoxifen in genomic low-risk patients (HR, 0.24; 95% CI, 0.10 to 0.60) and from goserelin in genomic high-risk patients (HR, 0.24; 95% CI, 0.10 to 0.54; Fig 3). Crude analysis yielded similar results (Data Supplement). No significant difference in patient and tumor characteristics was seen by trial arm and genomic risk group although a slightly younger age was observed in genomic low-risk tamoxifen-treated patients versus control (Data Supplement).

Compared with either therapy alone, further multivariable analyses showed no significant long-term benefit from the combination in genomic low-risk or high-risk patients (Table 2). However, in genomic high-risk patients, significantly increased long-term risk of distant recurrence from the addition of tamoxifen to goserelin was seen (HR, 3.36; 95% CI, 1.39 to 8.07). The interaction between goserelin and tamoxifen was significant in genomic high-risk patients (*P* = .006), but not in genomic low-risk patients (*P* = .080).

### Time-Varying Analysis of Endocrine Therapy Benefit

Time-varying multivariable analysis was conducted to assess how risk for distant recurrence and treatment benefit varies over time. This analysis focused on tamoxifen in genomic low-risk patients and goserelin in genomic high-risk patients, after the significant and potentially time-varying effects observed in the previous analyses.

Genomic low-risk patients had a steady long-term risk of distant recurrence. The estimated hazard rates were slightly increased from year 5 to year 10 and remained steady throughout the 20-year follow-up (Data Supplement). Moreover, a long-lasting benefit from tamoxifen was observed from year 4 to year 20 compared with control (Data Supplement) with significantly estimated HRs at years 5, 10, 15, and 20 showing a reduced risk (HR, 0.23; 95% CI, 0.06 to 0.92 at year 20; Table 3).

**TABLE 3.**
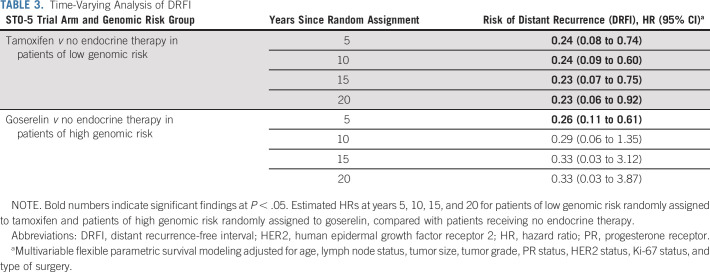
Time-Varying Analysis of DRFI

Genomic high-risk patients had early risk and early benefit from goserelin. A large increase in the hazard rates was observed within the first 5 years that rapidly decreased thereafter (Data Supplement). Significantly improved DRFI from goserelin was seen in the first 8 years compared with control (Data Supplement) with a statistically significant estimated HR of 0.26 (95% CI, 0.11 to 0.61) at year 5 (Table 3).

## DISCUSSION

This study of premenopausal patients with ER-positive breast cancer in a unique controlled randomization study demonstrates a significant 20-year benefit of 2 years of goserelin, tamoxifen, and the combination, compared with no adjuvant endocrine therapy. Furthermore, this study suggests that genomic low-risk patients have significant long-lasting benefit from tamoxifen, whereas genomic high-risk patients have early benefit from goserelin. The combination of goserelin and tamoxifen showed no long-term benefit over single treatment. Advantages of the STO-5 trial include long-term high-quality follow-up and inclusion of a control group of patients randomly assigned to no endocrine therapy.

The risk of distant recurrence and fatal metastatic disease in ER-positive patients remains steady decades after primary diagnosis, as observed by us^24,25^ and others.^21-23^ Thus, long-term follow-up in these patients is essential to understand the true treatment benefit. The mechanisms influencing late recurrences remain undefined, but potentially involve dormant low-proliferative tumor cells whose metastatic growth becomes awakened by undetermined microenvironmental or systemic stimuli, leading to the proliferation-apoptosis equilibrium later shifting in favor of metastatic proliferation.^20^

Genomic low-risk patients in the STO-5 trial have a steady long-term risk of distant recurrence, whereas genomic high-risk patients have high early risk, which demonstrates the heterogenous metastatic potential within premenopausal ER-positive patients. The observed differential treatment benefit suggests that 2 years of the ER agonist/antagonist tamoxifen more effectively prevents late recurrences in genomic low-risk patients, whereas 2 years of goserelin, inducing rapid systemic estrogen depletion, most effectively reduces the early risk in the more aggressive genomic high-risk tumors. These findings emphasize the clinical importance of biologic heterogeneity in ER-positive breast cancer and demonstrate the need to personalize premenopausal adjuvant endocrine therapy on the basis of prognostic and predictive tumor characteristics.

Today, the addition of OFS to tamoxifen is recommended to clinical high-risk patients, often defined by lymph node involvement, high grade, high proliferation, high genomic risk scores, or an age below 40 years.^6^ However, determining patients' risk is challenging and differs between studies. For instance, SOFT used a continuous composite risk measured from a Cox model including different tumor characteristics,^42^ whereas risk classification was performed by the combination of different tumor characteristics in the MINDACT trial.^43^ Here, we assessed patients' genomic risk using the 70-gene signature. As expected, high genomic risk is to a greater extent associated with more aggressive tumor characteristics, such as larger tumor size, higher grade, and HER2-positive status, but there is also clear interpatient heterogeneity. Notably, approximately 40% of genomic high-risk patients had lymph node–negative disease or low-proliferative tumors. Furthermore, according to the MINDACT definition,^43^ 22% of the STO-5 genomic high-risk patients were of low clinical risk. Clearly, a better understanding of risk is needed, especially the long-term risk (beyond 5-10 years) is largely unexplored,^44^ and risk definition will most likely differ between pre- and postmenopausal patients.

The STO-5 trial observed no benefit of the combination of goserelin and tamoxifen compared with single treatment and also suggests significant interaction between goserelin and tamoxifen. By contrast, SOFT showed improved disease-free and overall survival from the combination of OFS and tamoxifen compared with tamoxifen only, but not in the rate of distant recurrences.^10,11^ To note, random assignment to OFS only was not included in SOFT, and therefore, an interaction between OFS and tamoxifen could not be assessed. Also, the benefit from the combined therapy was mainly observed in younger clinically high-risk patients with regained premenopausal status after prior chemotherapy. Extended follow-up in SOFT will demonstrate whether there is significant long-term benefit of the combination versus tamoxifen only. Similar to our study, the addition of OFS to tamoxifen was not seen to improve survival in clinically defined low-risk patients in SOFT.^10,11^ Moreover, in a meta-analysis from 2007 and in a follow-up study of the ZIPP trials,^12,45^ no significant reduction in the risk of recurrence by the addition of goserelin to tamoxifen was observed.^46^

How goserelin and tamoxifen work on a mechanistic level, alone or in combination, is not fully understood. In brief, the initial response to goserelin is increased gonadotrophin-releasing hormone release in the hypothalamus. However, within a few weeks, desensitization occurs, and the ovarian estrogen production is inhibited, resulting in postmenopausal levels of circulating estrogens.^47,48^ On the other hand, tamoxifen acts as a mixed agonist/antagonist that when bound to the breast cancer ER alters its transcriptional activity and expression of proliferative-associated genes. In some tissues, including normal endometrium, tamoxifen acts primarily as an ER agonist, accounting for its association with endometrial cancer.^48-50^ Given the differences in mechanisms of action, we speculate that when tamoxifen and goserelin are given in combination, the agonistic effects of tamoxifen may counteract the estrogen-depleting effects of goserelin. Consistent with this, the ATAC trial suggested that tamoxifen's ER agonistic properties may counteract the simultaneous withdrawal of estrogen levels by anastrozole in postmenopausal patients.^13^

Patients in the STO-5 trial received 2 years of adjuvant endocrine therapy, but standard treatment nowadays is 5 years or more. The observed effect of 2 years of goserelin might be clinically important for patients who are unable to accept or endure 5 years of therapy, given the strong but reversible side effects associated with goserelin.^16-18^ However, further investigation is needed to understand potential differences between 2 and 5 years of therapy. Regarding tamoxifen, direct comparison between 2 years of 40 mg once daily as given in the STO-5 trial with today's at least 5 years of 20 mg once daily is difficult. The optimal dose and duration of endocrine therapy in premenopausal patients have not been established and may differ from that for postmenopausal patients.

There are limitations to this study. In the ER-positive subset of the STO-5 trial, sample size is a limitation and caution should be taken in the interpretation. Because of sample size, further subanalysis, for instance, on age was not statistically justifiable. Comparable with today's treatment approach, the STO-5 trial design allocated chemotherapy to lymph node–positive patients. Because of trial design, the additional effect from chemotherapy cannot be explored in the STO-5 trial. However, to note, 60% and 47% of genomic high-risk and low-risk patients, respectively, were lymph node–positive and received chemotherapy. Given the unique trial design and long-term follow-up by high-qualitative Swedish registries, results from this study are important to consider along with results from other trials. Importantly, no other studies on adjuvant endocrine therapy including OFS have reached more than a complete follow-up of 10 years or included a control group of patients randomly assigned to no endocrine therapy or random assignment to goserelin only.

In conclusion, results from the STO-5 trial with a 20-year follow-up suggest a long-term benefit from 2 years of adjuvant endocrine therapy in ER-positive premenopausal patients. Furthermore, long-lasting benefit from tamoxifen in genomic low-risk patients with steady long-term risk of distant recurrence is observed, whereas genomic high-risk patients with early risk benefit from goserelin. For patients unable to endure 5 years of endocrine therapy, the significant benefit from 2 years of treatment as seen in this study could be helpful for both patients and clinicians. However, further studies are needed to understand the optimal treatment duration. Moreover, no long-term benefit from combined goserelin-tamoxifen therapy over single treatment was observed in the STO-5 trial. This controlled randomization study has limited sample size, but allows unique assessment of the long-term effects of endocrine therapy in premenopausal patients.

## Data Availability

Restrictions apply to the availability of these data according to GDPR. Data were obtained from the STO Trialist Group and are available from the authors with the permission from the STO Trialist Group. Code availability: R-code to reproduce the results and figures are publicly available at https://github.com/annelieewa/STO5_20y_endocrine_therapy_benefit.
